# The new European guideline on cardiovascular disease prevention; how to make progress in general practice? Commentary on: Main messages for primary care from the 2016 European Guidelines on cardiovascular disease prevention in clinical practice

**DOI:** 10.1080/13814788.2017.1401063

**Published:** 2017-11-27

**Authors:** Suzanne Marchal, Arnoud W. J. van’t Hof, Monika Hollander

**Affiliations:** ^a^ Julius Centre for Health Sciences and Primary Care, University Medical Centre Utrecht Utrecht The Netherlands; ^b^ Department of Cardiology, Isala Zwolle The Netherlands; ^c^ Department of Cardiology, Maastricht University Medical Centre Maastricht The Netherlands; ^d^ Department of Cardiology Zuyderland Medical Centre Heerlen The Netherlands

**Keywords:** Prevention, general practice/family medicine, general, heart and circulation

## Abstract

The new guideline on cardiovascular disease (CVD) prevention, issued by the European Society of Cardiology was endorsed by 10 other societies, including Wonca Europe. It advices on how to reduce the cardiovascular (CV) risk in the population and attributes an important role to the general practitioner (GP). The GP is involved in treatment of the high-risk population as well as in public health measures to encourage a healthy lifestyle and CV risk factor reduction in the whole population. The new guideline gives room for a personalized approach and emphasizes that CV risk estimation and counselling need regular follow-up. We highlight the recommendations that most caught our eye and comment on the challenges for general practice.

KEY MESSAGES

- CVD prevention asks for a personalized approach, taking into account biological age, ethnicity, vitality, comorbidity and personal preferences.
- Estimation of the CV risk and assessment of lifestyle factors, including psychosocial aspects, should regularly be repeated.
- Depending on the healthcare system, GPs can play an essential role both in individual risk assessment and implementation of the guideline in national and regional prevention frameworks.
- CVD prevention needs to get a prominent place in clinical practice, supported by a clear policy and adequate organization.

## Commentary

### Risk estimation and personalized approach

In the new guideline [1], the accessible and simple to use risk chart to estimate the short-term (10 years) risk of cardiovascular death remains the basis for the total CV risk approach for prevention. Given that in this algorithm, age is the main driver of CV risk, young people may have many risk factors but almost never reach the treatment threshold, while most elderly have a high short-term risk based on age alone. A challenge is how to motivate young people for lifestyle change, even when the short-term risk is low. Fortunately, the new guideline provides practical tools that can help in the communication about CV risk and the urgency of lifestyle change, namely the relative risk score and ‘risk age.’ They can be used in all populations, irrespective of baseline risk. In the elderly, the new guideline recommends personalized care by taking into account quality of life, frailty or biological age. In general, more lenient treatment goals could be applied in elderly, for example, for systolic blood pressure or HbA1c. However, in vital elderly a more strict treatment regimen should be applied than in the frail. For the GP, guidance that is more practical is needed on how to assess and discuss vitality and personal preferences in the elderly [2].

Furthermore, attention is paid to the higher CVD risk in some ethnic minorities, survivors of cancer after treatment with chemotherapy or radiotherapy, women with a history of pre-eclampsia, pregnancy-induced hypertension, gestational DM, and/or a history of giving birth prematurely.

Besides, estimation of the CV risk should be repeated every five years. For individuals with risks close to treatment thresholds, an even more frequent CV risk assessment is recommended and patients with adverse lifestyle factors should regularly be counselled.

The new guideline continues to recognize the role of psychosocial stressors as CVD risk modifiers, barriers to treatment adherence and hampering factors for efforts to promote a healthy lifestyle. It provides a questionnaire to screen for psychosocial factors. However, implementation of this kind of tool in daily general practice has not been evaluated yet and will require sufficient consultation time and a plan to alleviate stress factors. Furthermore, referral options to social and psychological healthcare workers and support from local frameworks should be provided if indicated.

Altogether, it is a challenge for general practice to organize the care aligned to these recommendations. For the implementation of broad CVD prevention, adequate ICT support, as well as a trained multidisciplinary team, is essential including, e.g. GPs, practice nurses, dieticians, physiotherapists, psychologists and medical specialists. One of the challenges that lie ahead is how to exchange smoothly patient data and responsibilities in the healthcare chain.

### Role of measures of subclinical vascular disease in persons with moderate CV risk

The new guideline acknowledges that especially in patients with moderate risk who are near the treatment threshold, additional information on the presence of subclinical vascular diseases such as carotid plaques, coronary calcium score and the ankle–brachial index could be of help to reclassify the patient’s CV risk. However, systematically measuring these markers is not recommended since they have not yet been studied as screening tools. Further, clear thresholds above which a risk is substantially higher are also lacking.

In clinical practice, GPs are increasingly confronted with information on measurements that could reclassify someone’s risk. Weighing this information should be done with caution and should be done continuously at the discretion of the GP. It could be discussed in the shared decision-making process with the patient and/or the vascular specialist.

### Role of the GP and public health

Organizing broad CVD prevention is still a huge challenge in which the general practice plays an important role in promoting a healthy lifestyle across the population. It is important to realize that the responsibility of the GP extends beyond the clinical practice. What do GPs need to make it feasible? In addition, what is the role of the GP in unifying different stakeholders?

If a policy exists, GPs should have a role in integrating this policy into national and regional prevention frameworks. At the same time, the extra efforts of GPs in CVD prevention should be supported by concordant actions of surrounding organizations and (local) government and adequate finance. All involved organizations, including the government, local and regional authorities, and insurance companies need to take a stand on this issue. The guideline acknowledges that organizing CVD prevention is resource dependent.

A fundamental question to be answered is who is the problem-owner and who is responsible for orchestrating the process? To take responsibility, but also to explore borders, GPs have to outline a clear vision of their role in CVD prevention [3]. This vision may be dependent on the organization of healthcare in different countries.

### Evaluation of CVD prevention

Due to various causes, about half of the GPs use the European guidelines [4]. To improve the translation of the recommendations into clinical practice, the new guideline recommends monitoring and evaluating CVD prevention.

Furthermore, there are still gaps in the evidence for CVD prevention [5]. For example, the combined effect of the high-risk and population approach should be studied. Since this concerns a combination of two complex interventions, several methodological challenges will be encountered.

## Conclusion

The new European guideline on cardiovascular disease prevention provides room for improvement of CVD prevention in general practice. As we have to deal with an ageing population, an increasing prevalence of DM, a diverse target population for CVD prevention and the need for a more personalized approach and repeated CV risk-assessment of diverse target groups, GP’s will have to develop a vision on how to organize the practice providing the adequate care as is recommended in this guideline. GPs need to be proactive and need to collect data on possible CV risk factors systematically and routinely. Adequate ICT support with a reminder system should be considered and collaboration with other healthcare providers needs to be established.

GPs need to show leadership and to look over the walls of their practices to help build foundations for CVD prevention across the whole spectrum and to help promote a healthy lifestyle in the whole population. Finally, scientific evaluation of the effects of the combined high risk and population approach is needed for sustainable CVD prevention.
